# Antioxidant Activity of SOD and Catalase Conjugated with Nanocrystalline Ceria

**DOI:** 10.3390/bioengineering4010018

**Published:** 2017-02-25

**Authors:** Dmitry Gil, Jeannette Rodriguez, Brendan Ward, Alexey Vertegel, Vladimir Ivanov, Vladimir Reukov

**Affiliations:** 1 Department of Bioengineering, Clemson University, 301 Rhodes Hall, Clemson, SC 29634, USA; dgil@clemson.edu (D.G.); jeanner@g.clemson.edu (J.R.); bdward@g.clemson.edu (B.W.); vertege@clemson.edu (A.V.); 2 Kurnakov Institute of General and Inorganic Chemistry, Russian Academy of Sciences, Leninskii pr. 31, 119991 Moscow, Russia; 3 Institute for Biological Interfaces of Engineering, Clemson University, 301 Rhodes Hall, Clemson, SC 29634, USA

**Keywords:** nanoceria, antioxidants, superoxide dismutase

## Abstract

Interactions of nanoparticles with biological matter—both somatically and in nature—draw scientists’ attention. Nanoparticulate systems are believed to be our saviors, acting as versatile drug delivery vehicles. However, they can also cause life-threatening bodily damage. One of the most important properties of nanocrystalline cerium dioxide is its antioxidant activity, which decreases the abundance of reactive oxygen species during inflammation. In this paper, we report on synergistic effects of inorganic cerium oxide (IV) nanoparticles conjugated with the antioxidative enzymes superoxide dismutase and catalase on scavenging oxygen and nitrogen radicals.

## 1. Introduction

There is a growing body of evidence suggesting that oxidative stress plays a key role in the pathogenesis of numerous pathological conditions, including neurodegenerative diseases, acute lung injury and reperfusion injury, mood disorders, etc. [1,2]. In most cases, oxidative stress is primarily caused by a deficiency of antioxidants and/or an excess of reactive oxygen/nitrogen species (ROS/RNS) [3]. According to multiple reports in the literature, superoxide radicals ($\cdot O_{2}^{-})$, hydrogen peroxide ($H_{2}O_{2}$), hydroxyl radicals (·OH) and peroxynitrite (${\mathrm{ONOO}}^{-}$) cause the most harm in pathological conditions [2,4,5]. Besides causing oxidative injuries, intracellular reactive oxygen species are capable of triggering pro-inflammatory cytokine production [6]. Moreover, when released into the extracellular milieu, ROS may act as pro-inflammatory markers, worsening a pathological condition [7]. Thus, fighting oxidative stress, in particular the overproduction of ROS/RNS, appears to be of a great importance.

Over the past decades, multiple strategies to reduce the impact of oxidative stress have been developed. The use of naturally produced enzymes, such as superoxide dismutase (SOD) or catalase (CAT), or the genetic overexpression of these enzymes, has shown huge potential in in vitro and animal experiments [1,3,8,9]. In particular, attention has been focused on the effect of SOD therapy in myocardial reperfusion injury. Jolly et al. was the first to demonstrate the efficacy of superoxide dismutase in reducing the myocardial infarction size in animals [10]. That study was followed by a number of reports showing SOD’s beneficial effect in experimental models of different ischemia-reperfusion injuries [11,12,13]. Moreover, it was shown that SOD therapy can be successfully used following radiotherapy to reduce the concentration of ROS [14]. Targeted delivery of antioxidant enzymes, including SOD and CAT, appears to be of particular interest. Multiple strategies have been developed in an attempt to deliver the enzymes directly to certain types of cells, or even to the specific organelles [15,16,17,18].

At the same time, considerable evidence suggests either a failure of SOD to protect from oxidative damage or a drop in therapeutic efficacy with increased duration of reperfusion. It has been suggested that the great disparity in the results of SOD therapy is related to either the dose of the enzyme tested or the experimental conditions of the study [19,20]. Another theory suggests that the lack of reproducibility of SOD therapy trials could be caused by enzyme inhibition by peroxide radicals and/or hydrogen peroxide formed as the result of superoxide radical dismutation [21].

A possible solution could be the concurrent use of SOD and catalase—another vital component of biological defense against oxidative stress [22]. CAT facilitates the decomposition of hydrogen peroxide into water and oxygen. However, the activity of catalase itself can be drastically reduced in the presence of superoxide radicals, the generation of which can reach up to 3 nmol/min under pathological conditions [23,24]. Moreover, in order to create a mutually protective set of SOD and CAT, the ratio of the enzymes should be precisely controlled, which can be performed by cross-linking the enzymes with glutaraldehyde [25]. However, there are several concerns regarding glutaraldehyde stability in vivo [26]. Therefore, although the SOD-CAT therapy was found to be fairly effective, there is still enough room for improvement. In the present work we propose a novel approach to address the issues raised above with the conjugation of SOD and CAT to nanocrystalline cerium dioxide, known as an efficient ROS scavenger.

Cerium dioxide (ceria) is a multifunctional material known for its high catalytic performance in various applications [27]. Most of ceria’s unique properties are primarily due to the presence of mixed valence states of Ce^3+^ and Ce^4+^ and the presence of oxygen vacancies [28]. In particular, partial reduction of Ce^4+^ to Ce^3+^ on the surface gives rise to ceria’s pronounced antioxidant properties. In the nanocrystalline state, due to the large surface-to-volume ratio, ceria exhibits an abundance of oxygen vacancies with relatively high mobility [29]. The resulting strong nonstoichiometric properties of nanocrystalline ceria (nanoceria) make it a potent material for biomedical application. Of particular interest is the ability of nanoceria to protect against oxidative stress. Multiple animal and cell culture studies have demonstrated that nanocrystalline cerium dioxide is an effective ROS scavenger [30]. Moreover, it was shown that nanoceria can act as a SOD or CAT mimetic, and the efficacy of the compound to scavenge radicals is directly related to the concentration of Ce^3+^ ions on the surface of the particles [28,31]. These studies suggest that nanoceria is capable of neutralizing superoxide radicals and hydrogen peroxide—two compounds that inhibit the activity of catalase and superoxide dismutase.

Previously we demonstrated that enzymes such as superoxide dismutase and catalase can be conjugated with nanoparticles without the loss of enzymatic activity [32]. The present work focuses on the conjugation of SOD and CAT to nanocrystalline cerium dioxide in an attempt to create a robust antioxidant system.

## 2. Materials and Methods

### 2.1. Materials

Cu/Zn SOD (from bovine erythrocytes), catalase (from bovine liver), cerium (III) nitrate, ammonia, citric acid, SOD assay kit (Cat # 19160) and lipopolysaccharides (LPS) were obtained from Sigma-Aldrich (St. Louis, MO, USA). Amplex^®^ Red catalase assay kit (Cat # A22180) was purchased from Thermo Fisher Scientific (Waltham, MA, USA). Dulbecco's Modified Eagle Medium (DMEM), fetal bovine serum (FBS), penicillin and streptomycin were purchased from Corning Inc. (Manassas, VA, USA). RAW 264.7 (ATCC^®^ TIB-71™) cell line was obtained from ATCC^®^ (Manassas, VA, USA). Griess reagent was obtained from Enzo Life Sciences (Farmingdale, NY, USA).

### 2.2. Synthesis of Nanocrystalline Ceria

Cerium dioxide colloidal solution was synthesized according to the method described elsewhere [33]. Briefly, 25 mL of the aqueous solution of 0.4 M cerium (III) nitrate containing 2.0 g of citric acid was prepared and then mixed with 100 mL of a 3 M ammonia solution under mild steering. The resulting colloidal solution was kept at RT for 2 h for the formation of CeO_2_ to be fully completed. Then, the sols were rinsed by copious amounts of deionized water.

### 2.3. Physicochemical Characterization of Ceria Nanoparticles

Phase composition and cell parameter of the obtained ceria were determined by means of X-ray powder diffraction (XRD). These measurements were performed using Rigaku D/MAX 2500 (Rigaku, Woodlands, TX, USA). Diffraction peaks were identified with JCPDS data base. Following cell parameter calculations (*a*), the nonstoichiometric parameter (***δ***) was determined according to the empirical formula [33]:

*а* (nm) = 0.5413 + 0.04612* ***δ***

The morphology of ceria nanoparticles was assessed by means of transmission electron microscopy (TEM) (Hitachi H9500, Schaumburg, IL, USA). Images of the samples were obtained with an accelerating voltage of 100 kV. Additionally, ceria particle size was determined using dynamic light scattering (DLS) (Brookhaven 90 plus, Holtsville, NY, USA). The concentration of ceria in the obtained soles was determined by thermogravimetric analysis (TGA) (TA instruments, New Castle, DE, USA).

### 2.4. Preparation of Ceria-Enzyme Conjugates

The conjugates were prepared according to the methodology adapted from elsewhere [34]. Briefly, aqueous solution containing SOD or CAT with the concentrations of 1 U/mL for each of the enzymes was mixed with the ceria sol; the resulting solution was incubated at RT for 2 h.

### 2.5. Assessment of Antioxidant Activity

The antioxidant activity of the conjugates against superoxide radicals was assessed by the SOD enzymatic assay. It was performed using WST-1 reagent as an indicator dye; superoxide radicals were generated by the xanthine/xanthine oxidase (0.25 U) reaction. In its turn, the ability of the conjugates to scavenge hydrogen peroxide was evaluated using catalase assay (Amplex^®^ Red assay). The concentration of hydrogen peroxide used in the assay was 2 μM, which is relevant to the physiological level [31].

Additionally, antioxidant activity of the conjugates was measured in the cell culture experiments. These studies were conducted according to the protocol adapted from elsewhere [35]. Briefly, prior to the experiments mouse macrophages (RAW 264.7, ATCC^®^ TIB-71™) were cultured at 37 °C under 5% CO_2_ until reaching 80% confluency. Then the cells were passaged, diluted and 1 × 10^5^ macrophages were seeded in a sterile 24-well plate and incubated overnight at 37 °C under 5% CO_2_ to allow cell attachment. Following incubation, aliquots of the samples were added to the wells and kept at 37 °C for 4 h. Then, the cells were LPS challenged (10 μg/mL) and incubated for another 4 h at 37 °C under 5% CO_2_. Afterwards, the samples were treated with an equal amount of Griess reagent. Following 30 min incubation, the optical density of the resulting solution was recorded at 540 nm.

## 3. Results and Discussion

Over the past years, numerous synthetic approaches for the preparation of nanoceria have been developed. However, the potential use of cerium dioxide in biomedical applications puts certain constraints on its formation method. In particular, the compound should be stable to aggregation and appropriate for dosing, biocompatible, and the particle size should not exceed 2–3 nm to achieve pronounced antioxidant properties [36]. In an attempt to meet these requirements, we synthesized stable ceria colloidal solutions stabilized by citric ions.

**Figure 1:**
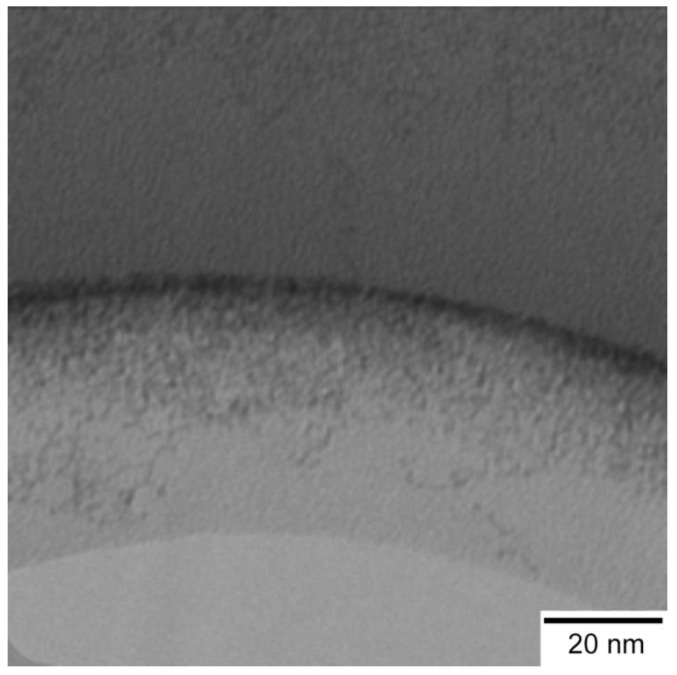
Transmission electron microscopy images of ceria sols.

The obtained ceria sols were dried and then characterized by a number of physicochemical techniques. The XRD results showed that the proposed methodology allowed us to prepare cubic ceria with a cell parameter of ~5.442 Å, suggesting the stoichiometry of the samples to be CeO_1.90_ [27]. The results of TEM demonstrated that the particle size was in the range of 2–3 nm (see Figure 1).

Little or no evidence of particle aggregates was found on the micrographs. These results were further confirmed by dynamic light scattering, which showed that hydrodynamic diameter of the ceria nanoparticles in the sols was 2.3 ± 1 nm. The stability of the sols was confirmed by long-term storage experiments, which showed no observable precipitation over the period of six months. Therefore, the synthetic approach used in this study resulted in stable ceria colloidal solutions with a particle size small enough that pronounced antioxidant properties could be expected. The concentration of ceria in the obtained samples was determined by thermogravimetric analysis and was calculated to be 0.01 M. At this concentration, the antioxidant effect of ceria nanoparticles is expected to occur [37,38].

The antioxidant activity of the conjugates was assessed by means of SOD and catalase enzymatic assays. Additionally, cell culture experiments with lipopolysaccharide-challenged macrophages were conducted to evaluate the ability of the conjugates to scavenge extracellular radicals. The obtained results were compared with those for the non-functionalized ceria nanoparticles and SOD/CAT.

**Figure 2:**
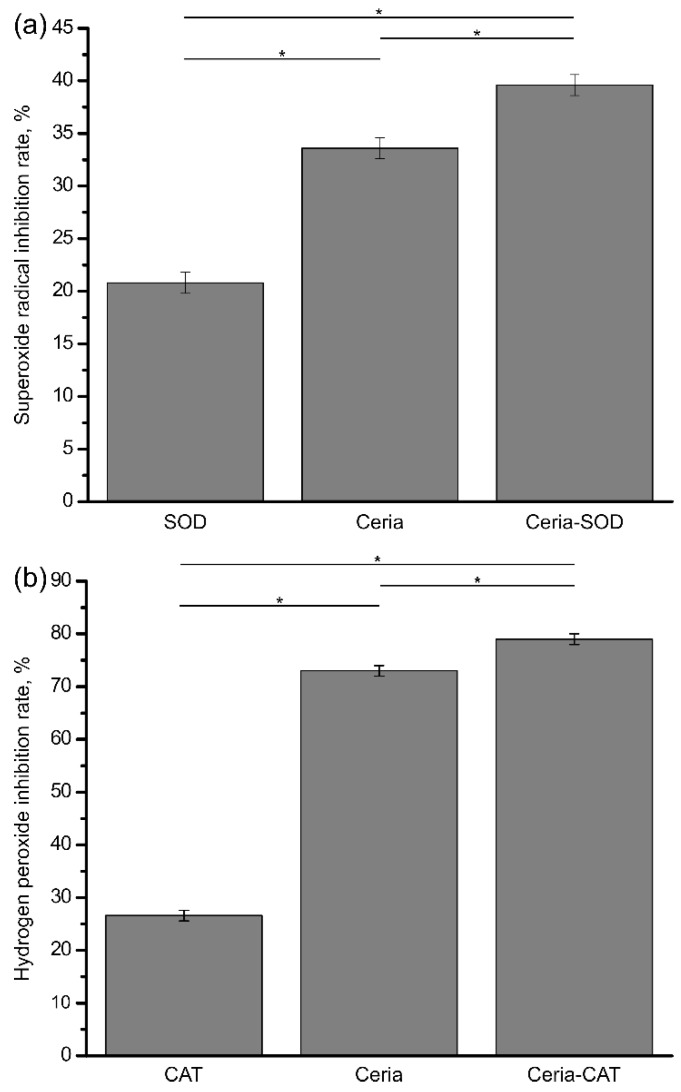
The results of the SOD enzymatic assay (**a**) and Amplex^®^ Red assay (**b**) performed for SOD-ceria and CAT-ceria conjugates, non-functionalized ceria, catalase and superoxide dismutase. Based on the obtained calibration curves, the percentage of superoxide radicals (**a**) and hydrogen peroxide (**b**) inhibition results were calculated and are presented herein. Asterisks represent significant difference between values (*p* < 0.05, *n* = 4).

The results of the SOD enzymatic assay (Figure 2a) showed that the conjugates exhibited excellent SOD mimetic activity, effectively scavenging superoxide radicals. Moreover, the SOD mimetic activity of the ceria-SOD conjugates was found to be significantly higher than that of the unfunctionalized ceria nanoparticles and pure SOD (*p* << 0.001, *n* = 4).

The ability of the conjugates to scavenge hydrogen peroxide was evaluated using the catalase assay (Amplex^®^ Red assay). The results are shown in Figure 2b. The ceria-CAT conjugates demonstrated pronounced catalase mimetic activity against the physiologically relevant hydrogen peroxide concentration of 2 μM [31]. Again, the activity of the conjugates was found to be significantly higher than that of pure nanoceria and catalase (*p* = 0.001, *n* = 4).

Therefore, the obtained results are indicative of the synergetic effect of nanocrystalline ceria and superoxide dismutase or catalase absorbed on its surface.

In the present study the conjugates’ antioxidant activity against extracellular reactive nitrogen species was also evaluated. Nitric oxide (NO) plays an important role in numerous physiological processes including oxidative stress. Generated and released by macrophages and neutrophils, NO is able to kill viruses and pathogenic bacteria and even to act as an ROS scavenger [39]. However, when exposed to superoxide radicals, nitric oxide is also involved in formation of peroxynitrite (${\mathrm{ONOO}}^{-}$) and hydroxyl radicals (·OH), both of which appear to be cytotoxic agents:

$$\mathrm{NO}+\cdot O_{2}^{-}\to {\mathrm{ONOO}}^{-}{+H}^{+}\to \cdot \mathrm{OH}+\cdot {\mathrm{NO}}_{2}$$

**Figure 3:**
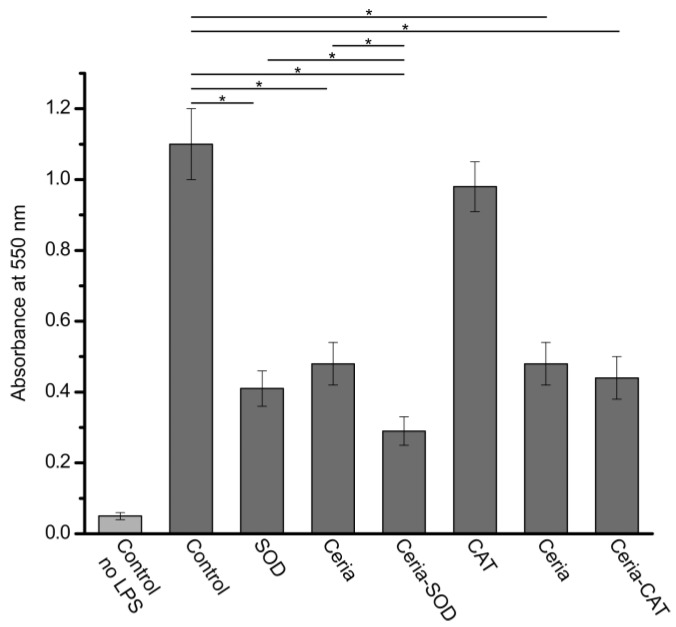
The results of the Griess test conducted for SOD- and CAT-ceria conjugates, and non-functionalized ceria, superoxide dismutase and catalase. The measured absorbance at 550 nm is directly correlated with the content of $\cdot {\mathrm{NO}}_{2}$ in the cell culture media. Asterisks represent significant difference between values (*p* < 0.05, *n* = 4).

Given the excellent antioxidant activity of the ceria-SOD/CAT conjugates against ROS, one would expect to see efficacy in eliminating peroxynitrite radicals. To prove this hypothesis, the activity of the conjugates against RNS was tested in the cell culture experiments with mouse macrophages challenged by LPS. It was shown that upon exposure to LPS, macrophages release a milieu of oxidants and enzymes, including different RNS [40]. The Griess test was used to determine the content of the RNS (Figure 3). As would be expected, SOD-ceria conjugates possessed pronounced scavenging activity, effectively reducing the concentration of $\cdot {\mathrm{NO}}_{2}$. Moreover, the efficacy of the SOD-ceria conjugates was significantly higher than that of individual nanoceria and SOD (*p* << 0.001, *n* = 4).

In the case of the CAT-ceria conjugates, the measured antioxidant activity appeared to be lower than that of the SOD-ceria conjugates and remained on the level of pure nanoceria. CAT alone had a relatively low scavenging efficacy. These results were not unexpected given the antioxidant mechanism of catalase. At the same time, the antioxidant activity of the CAT-ceria conjugates was apparently dictated by the RNS-scavenging properties of nanoceria.

In the present study, the antioxidant properties of ceria conjugates were tested in the presence of LPS-challenged macrophages. The pronounced tendency of activated macrophages to phagocytose foreign objects could have caused elevated cellular uptake of the conjugates, increasing the concentration of the antioxidants in intracellular space. Therefore, the measured antioxidant activity of the conjugates can be expected to be lower when determined in the presence of unactivated macrophages/monocytes. On the other hand, in the experiments with nanoceria it was shown that cellular uptake is more pronounced for 100 nm particles. In the case of smaller particles (3 nm), the cellular uptake was found to be significantly lower [41]. However, more detailed cell culture studies are required to evaluate cellular uptake and to determine the ability of the conjugates to scavenge intracellular ROS and RNS.

Given the excellent antioxidant properties of ceria conjugates studied in the present work, one can envision potential medicinal and clinical applications of the proposed antioxidant system. There is a number of in vivo studies showing the efficacy of nanocrystalline ceria in the treatment of different pathological conditions, including reproductive, neurological, gastrointestinal, ophthalmologic, etc. [36,42,43]. Taking into account the significantly higher antioxidant activity of the conjugates analyzed in this study, their efficacy in vivo can be expected to be even higher than that of unmodified ceria. However, in order to discuss these findings within the scope of potential biomedical applications, it is imperative to assess the distribution of the conjugates in vivo, as well as their toxicity and stability. Thus, thorough animal studies are required to prove that the potential benefits of the use of ceria conjugates outweigh the complexity and possible adverse effects of the therapy.

## 4. Conclusions

The present study deals with antioxidant conjugates based on nanocrystalline ceria and superoxide dismutase or catalase. In in vitro experiments, we showed that SOD/CAT and nanoceria were capable of complementing each other, providing a synergetic effect towards antioxidant activity. The cell culture experiments provided additional evidence of the pronounced antioxidant activity of the conjugates. This work is a foundation for more detailed in vitro, cell culture and animal studies.
